# Association between multimorbidity and undiagnosed obstructive sleep apnea severity and their impact on quality of life in men over 40 years old

**DOI:** 10.1017/gheg.2018.9

**Published:** 2018-06-04

**Authors:** G. Ruel, S. A. Martin, J.-F. Lévesque, G. A. Wittert, R. J. Adams, S. L. Appleton, Z. Shi, A. W. Taylor

**Affiliations:** 1 Direction québécoise du cancer, Ministère de la Santé et des Services sociaux, Québec, Canada; 2 Centre de recherche du CHUM, Université de Montréal, Montréal, Canada; 3 Discipline of Medicine, University of Adelaide, South Australia, Australia; 4 Bureau of Health Information, New South Wales, Australia; 5 Health Observatory, The Queen Elizabeth Hospital, Woodville, South Australia, Australia; 6 Population Research and Outcome Studies, University of Adelaide, South Australia, Australia

**Keywords:** Apnea-hypopnea index, chronic disease, comorbidity, obstructive sleep apnea, quality of life

## Abstract

**Background.:**

Multimorbidity is common but little is known about its relationship with obstructive sleep apnea (OSA).

**Methods.:**

Men Androgen Inflammation Lifestyle Environment and Stress Study participants underwent polysomnography. Chronic diseases (CDs) were determined by biomedical measurement (diabetes, dyslipidaemia, hypertension, obesity), or self-report (depression, asthma, cardiovascular disease, arthritis). Associations between CD count, multimorbidity, apnea-hyponea index (AHI) and OSA severity and quality-of-life (QoL; mental & physical component scores), were determined using multinomial regression analyses, after adjustment for age.

**Results.:**

Of the 743 men participating in the study, overall 58% had multimorbidity (2+ CDs), and 52% had OSA (11% severe). About 70% of those with multimorbidity had undiagnosed OSA. Multimorbidity was associated with AHI and undiagnosed OSA. Elevated CD count was associated with higher AHI value and increased OSA severity.

**Conclusion.:**

We demonstrate an independent association between the presence of OSA and multimorbidity in this representative sample of community-based men. This effect was strongest in men with moderate to severe OSA and three or more CDs, and appeared to produce a greater reduction in QoL when both conditions were present together.

## Introduction

Obstructive sleep apnea (OSA) is defined as the cessation of naso-buccal air flow for more than 10 s [1] and is diagnosed based on the apnea-hypopnea index (AHI) [2]. A systematic review [3] showed the impaired quality of life (QoL) in OSA patients when compared with population norms. Severe OSA has also been demonstrated to increase all-cause mortality [4]. Risk factors for OSA include ageing, elevated Body Mass Index (BMI) and male gender [5, 6]. Aside from the well-known relationship with obesity, OSA has been previously reported to be associated with hypertension [7], cardiovascular disease (CVD) [7, 8], diabetes [9, 10], chronic obstructive pulmonary disease [11] and depression [11].

Multimorbidity, or the presence of two or more medical conditions in an individual [12], has a high prevalence in numerous populations [13–15]. Multimorbidity prevalence increases with age [14, 16] and social deprivation [16, 17], and is associated with increased medical consultations, prescriptions, emergency hospital utilisation, hospital length of stay and mortality rates [18–20]. The relationship between the presence of OSA and the development of multimorbidity and the subsequent impact on QoL remains unclear [11, 21]. This is complicated by the fact that OSA remains a severely under-diagnosed condition, particularly in middle-aged to elderly men. Recent estimates suggest approximately 75–80% of OSA cases may be undiagnosed [22, 23] thereby limiting any understanding of the true association between OSA and chronic disease burden. High multimorbidity has been previously demonstrated in men with severe but not moderate or mild OSA [21] among a primary care population referred to a sleep laboratory for further evaluation. To the best of our knowledge, there is no information on the relationship between multimorbidity and OSA among the general population. Consequently, our aim was to examine whether there is an independent association between the presence and severity of OSA and multimorbidity, and subsequent impacts on QoL, in a community-based cohort of middle-aged to elderly men.

## Methods

### Study design

**Fig. 1.**
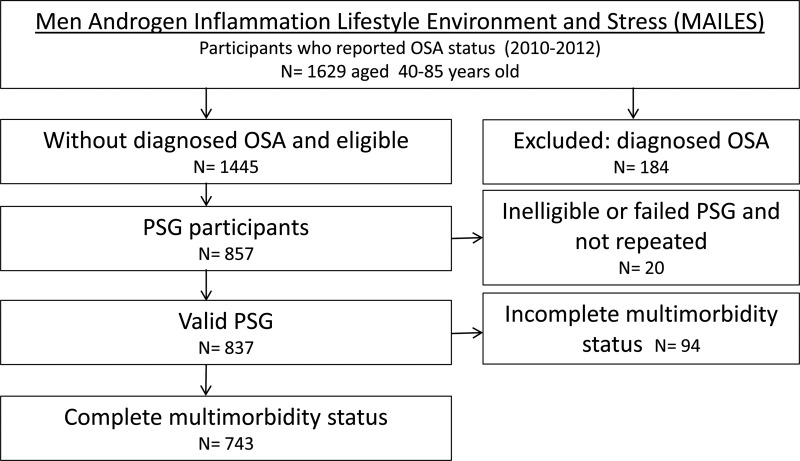
Flow chart of the sleep substudy of the MAILES cohort. OSA, obstructive sleep apnea; PSG, polysomnography; and AHI, apnea hypopnea index.

The Men Androgen Inflammation Lifestyle Environment and Stress (MAILES) Study comprises randomly selected urban community-dwelling men from South Australia aged 35 years at the time of enrolment [24]. Initial random sample selection was by Electronic White Pages and recruitment by computer-assisted telephone interviews (CATI) occurred in 1999–2005. Biomedical, health, lifestyle and behavioural assessment was conducted in two hospital-based clinics at baseline and follow-up (2008–10), using standardised and reproducible study protocols including anthropometry. Of the 2038 participants at follow-up, 1445 without previously diagnosed OSA were invited to participate in a sleep substudy with 75.2% agreeing. Of these, a random sample of 1000 men was chosen for inclusion and by the end of the study period, 837 successfully underwent polysomnography (PSG). For the current analysis, we excluded men with incomplete information for any CD leading to a total of 743 men (Fig. 1). Previous analyses have demonstrated that the 837 men who participated in the MAILES sleep study were broadly representative of the target population [24]. An additional analysis of these men and the current analytic sample, demonstrated comparability across age group, education, region of birth, marital status, with the current analytic sample have a slightly higher proportion of men from higher income households and retirees (data not shown).

The study was approved by Queen Elizabeth and the Royal Adelaide Hospital Human Research Ethics Committees (HREC) and written consent were obtained.

### Obstructive sleep apnea

In 2010–2011, subjects without a previous OSA diagnosis underwent 8-channel in-home unattended PSG (Embletta X100, Embla Systems, Colorado) which measured Electroencephalogram (EEG), Electroculogram, Electromyogram, nasal pressure, thoracic and abdominal effort, oximetry, body position, and limb movements. Trained staff visited study participants in their homes to set-up and attach the sleep study equipment, and measure neck circumference. Failed studies were repeated if possible. A single experienced sleep technician performed manual scoring of all home PSGs according to the 2007 American Academy of Sleep Medicine (AASM) (alternate) criteria [25]. An apnea was defined as cessations of nasal flow lasting ⩾10 s, and hypopneas as a >50% decrease in nasal flow (or in both thoracic and abdominal excursions) and associated ⩾3% oxygen desaturation or an EEG arousal. OSA was defined as an AHI ⩾10/h. Prior work suggests an AHI of 5/h used to define sleep-disordered breathing in the Wisconsin study [2] is approximately equivalent to an AHI of 10/h using the alternate AASM definition [26]. The Epworth Sleepiness Scale [27], Pittsburgh Sleep Quality Index [28] and the STOP questionnaire [29] were administered at the time of the PSG.

### Multi-morbidity and chronic disease assessments

Multimorbidity was defined as per [12] as the concurrent presence of two or more of the following nine conditions assessed at study follow-up clinics (2008–10): asthma, heart disease, diabetes, depression, hyperlipidemia, hypertension, obesity, osteoarthritis and rheumatoid arthritis. Depression, diabetes, hyperlipidemia, hypertension and obesity were determined during the clinic visit (see [30] for an overview). Height was measured to the nearest 0.5 cm using a stadiometer, and weight to the nearest 0.1 kg in light clothing and without shoes using standard digital scales. BMI was calculated as weight (kg)/height (m)^2^. Obesity was defined as a BMI ⩾30 kg/m^2^. A fasting blood test was performed to determine total cholesterol and fasting plasma glucose (FPG) levels. Men were deemed to have diabetes if they self-reported having ever been told by a doctor they had the condition, self-reported utilisation of glucose-lowering drugs or by measurement at the clinic (FPG⩾7.0 mmol/L) [31, 32]. Hyperlipidemia was defined as total blood cholesterol ⩾5.5 mmol/L or self-report use of cholesterol-lowering drugs [33]. Three blood pressure measurements were taken 5–10 minutes apart using a standard, calibrated blood pressure sphygmomanometer, while the participant was relaxed and seated. The average of the final two measures was used in the analyses. High blood pressure was defined as systolic blood pressure⩾140 mm Hg and/or diastolic blood pressure⩾90 mm Hg or the self-reported use of drugs to lower blood pressure. Depression status was determined using either a score over 15 for the Centre for Epidemiological Studies Depression Scale [34] or a score over 13 for the Beck Depression Index [35]. Participants self-reported if they had ever been told by a doctor they had asthma, any heart condition (including stroke), osteoarthritis or rheumatoid arthritis.

### QoL assessment

Health-related QoL was assessed using the Short Form 36 questionnaire (SF-36) at study follow-up. The first general health questions (SF1), as well as the mental and physical component summary (MCS and PCS) domains, scored on Australian normative data, were used in the analysis [36].

### Physical and socio-demographic characteristics

Participants were interviewed by trained health workers using a pre-coded questionnaire at study follow-up. Individual-level variables included age, gender, marital status and education level, household income, receiving government benefits or pension and employment status. Detailed demographic, biographical and risk factor information was collected via a self-completed questionnaire.

### Statistical analyses

Initial descriptive analyses were conducted to examine the correlation between the outcome measure (multimorbidity; expressed initially as the total number of CDs) and exposure measure (OSA; expressed as continuous AHI), and selected physical characteristics, socio-demographic factors, CD and QoL status. Spearman correlation coefficients (*r*_*s*_) were calculated to approximate the direction and degree of the monotonic relationship between AHI and CD values, and with the selected covariates. Participants were grouped across the four exposure levels [absent/none (AHI<10), mild (AHI ⩾10 and <20), moderate (AHI⩾20 and <30) and severe (AHI⩾30)] to compare against the number of CDs and QoL (PCS and MCS scores of the SF-36) using the Kruskal–Wallis *H* test. Multinomial logistic regression models for multimorbidity (2+ CDs *vs* 0–1 CD & 3+ CDs *vs* 0–2 CDs) and QoL were then fitted across all exposure levels (Absent, Mild, Moderate and Severe) to determine crude, age-adjusted, and multi-adjusted odds ratios. Finally, to test for any interaction between multimorbidity and OSA on QoL, number of CDs, OSA severity categories and their interaction terms were compared against the PCS and MCS domains of the SF-36. (as measured by the SF-1, MCS and PCS). Unless stated otherwise, standard errors are presented in tables and standard deviation in the text. Crude data are presented in tables and text. All analyses were carried out using SPSS version 20.0 (SPSS, Inc., Chicago, IL, USA) and a *p* < 0.05 was considered significant.

## Results

Table 1 describes the baseline physical and socio-demographic characteristics of the 743 study participants. The mean age of the cohort was 59 ± 11 years (mean age ± s.d.) and the range was between 41 and 87 years. The mean BMI was 28.6 ± 2.6 kg/m^2^ and 44% of the cohort were obese (BMI⩾30 kg/m^2^). Fifty-two percent of the cohort had OSA as defined by an AHI value over 10.

**Table 1.** Baseline physical and socio-demographic characteristics of the 743 participants

Variable	*N* (%)
Age groups	
40–49	186 (25)
50–59	230 (31)
60–69	195 (26)
70+	132 (18)
Education	
Secondary/refused	2342 (32)
Trade/Apprenticeship	173 (23)
Certificate/Diploma	216 (29)
Bachelor degree or higher	112 (15)
Occupation	
Full time employed	416 (56)
Part time/casual employment	61 (8)
Retired	217 (29)
Unemployed	13 (2)
Home duties/student/other/not stated	31 (4)
Annual Income (Australian $)	
Up to $ 12 000	15 (2)
$ 12 001–$ 20 000	50 (7)
$ 20 001–$ 40 000	149 (20)
$ 40 001–$ 60 000	153 (21)
$ 60 001–$ 80 000	107 (14)
Over $ 80, 000	232 (31)
Not stated	37 (5)
Marital status	
Married/living with partner	592 (80)
Separated/divorced	74 (10)
Widowed	31 (4)
Never married	43 (6)
Not stated	3 (0)
BMI status	
Underweight/normal (−25)	186 (25)
Overweight (25–29)	230 (31)
Obese I (30–34)	195 (26)
Obese II+ (35+)	132 (18)
Physical activity	
Sedentary	171 (23)
Low activity	314 (42)
Moderate activity	188 (25)
High activity	62 (8)
Not stated	8 (1)
Smoking status	
Yes/occasionally	119 (16)
No	624 (84)

AHI was shown to have a comparable, positive correlation coefficient with multimorbidity status and CD count, as with neck circumference and age; both recognised risk factors for OSA. CD count and AHI were both negatively correlated with general QoL and the PCS domain of the SF-36, and to a lesser degree the MCS, with CD count demonstrating a significantly stronger correlation with all measures QoL indices. AHI and number of CDs also showed a positive correlation between work and smoking status and negative correlation with income status (with higher CD count additionally associated with lower education status) (Table 2).

**Table 2.** Bivariate associations between AHI and chronic disease count and physical, social, quality of life and sleep questionnaire

	AHI		CD Count	
	R*	*p* value	R*	*p* value
Multimorbidity				
Multimorbidity status	0.22	*p* ⩽ 0.001	NA	NA
CD count	0.26	*p* ⩽ 0.001	NA	NA
Physical characteristics				
Neck circumference	0.27	*p* ⩽ 0.001	0.37	*p* ⩽ 0.001
Age	0.20	*p* ⩽ 0.001	0.21	*p* ⩽ 0.001
Social characteristics				
Work status	0.18	*p* ⩽ 0.001	0.14	*p* ⩽ 0.001
Smoking status	0.08	*p* ⩽ 0.05	0.07	*p* ⩽ 0.05
Marital status	0.02	ns	0.07	NS
Physical activity	−0.01	ns	0.02	NS
Education	−0.06	ns	−0.11	*p* ⩽ 0.05
Income status	−0.11	*p* ⩽ 0.005	−0.13	*p* ⩽ 0.001
Quality of life (SF−36)				
General health status (SF1:1–5)	−0.12	*p* ⩽ 0.001	−0.25	*p* ⩽ 0.001
Physical component summary (PCS)	−0.10	*p* ⩽ 0.005	−0.24	*p* ⩽ 0.001
Mental component summary (MCS)	−0.04	NS	−0.19	*p* ⩽ 0.001

*Spearman correlation coefficient.

**Fig. 2.**
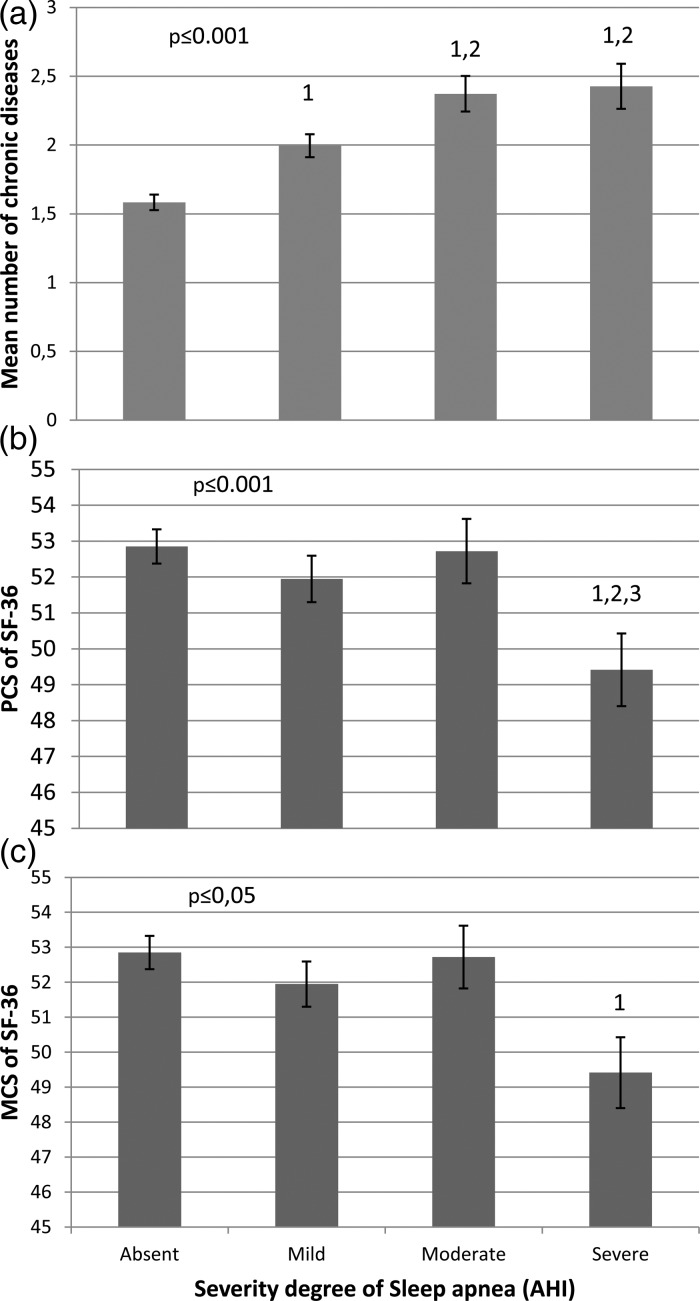
Relationship between obstructive sleep apnea severity and the mean number of chronic disease and PCS and MCS SF-36 score in the 743 men. ^1, 2 and 3^, significantly different from the absent, mild and moderate severity group, respectively. Data are adjusted for age, sedentary lifestyle, marital, gross income, working and smoking status.

Among those with OSA, 52, 27 and 21% of the population had mild (AHI⩾10 and <20), moderate (AHI⩾20 and <30) and severe (AHI⩾30) OSA, respectively. The mean number of CDs increased progressively for those with mild and moderate OSA severity and plateaued thereafter (Fig. 2, panel A). Overall, men in the severe group had 0.78 more CDs [95% CI (0.42–1.14)] than those without OSA (*p* ⩽ 0.001 in the fully adjusted model). AHI was 9.4 events per hour higher in those with three or more CDs than those without any CD (*p* ⩽ 0.001).

Among the 743 men included, 58% of the population had multimorbidity. The proportion of those with multimorbidity was higher in those with OSA than in those without (67% *v.* 48%, *p* ⩽ 0.001). There was no significant difference in multimorbidity proportion between the mild, moderate and severe groups, although the mean number of CDs was significantly higher in the moderate and severe groups when compared with the mild severity group (Fig. 2). However, when the moderate and severe groups were pooled, the proportion of those with three CDs in this group was 12% higher (*p* ⩽ 0.05) than those in the mild group, suggesting that those with three CDs have a major impact on the relationship between multimorbidity and the severity of OSA. Among individual CDs, the proportions of hypertension, obesity, diabetes and CVD were 22% (*p* ⩽ 0.05), 28% (*p* ⩽ 0.05), 8% (*p* ⩽ 0.05) and 10% (*p* ⩽ 0.05) higher in those with moderate or severe OSA than in those without OSA. The proportions of those with hypertension and depression were 14% (*p* ⩽ 0.05) and 7% (*p* ⩽ 0.05) higher in those with mild OSA than in those without the disease. Obesity was the only CD for which there was a significant difference between the mild and the moderate/severe group (+20%, *p* ⩽ 0.05). There was also a detrimental impact of OSA on both the PCS (*p* ⩽ 0.0001) and MCS (*p* ⩽ 0.05) summary scores of QoL but only at a severe OSA level.

Odds ratios for OSA severity status were examined according to the presence of multimorbidity (Table 3). When compared with those without, those with multimorbidity were more likely to have mild, moderate or severe OSA than not having OSA. More precisely, the unadjusted odds ratios of having mild, moderate or severe OSA were 1.91 (1.34–2.72), 2.92 (1.80–4.73) and 2.52 (1.51–4.21), respectively, when compared with those without OSA. The odds ratios for those with more than three CDs were 3.27 (1.47–7.29), 5.11 (2.17–12.04) and 6.59 (2.78–15.64) for the mild, moderate and severe OSA groups when compared with those without OSA. The odds ratio of having fair or lower health status was also significantly higher in those with severe OSA than in those without the condition [3.20 (1.72–5.93)].

**Table 3.** Differences in proportions of the participants and estimated odds ratios between OSA severity categories for multimorbidity, quality of life, sleepiness and sleep quality and estimated OSA risk

	OSA status				Model	
	Absent	Mild	Moderate	Severe	Unadjusted	Adjusted for age & social
*N*	360	199	102	82		
Multimorbidity (2+) *v.* no (0–1)					*p* ⩽ 0.001	*p* ⩽ 0.001
Proportion 2+ (*n* (%))	171 (48)	126 (63)^a^	74 (72)^a^	57 (70)^a^		
Odds Ratio*	1	1.72	2.68	2.02		
(95% CI)		(1.19–2.72)	(1.63–4.42)	(1.19–3.44)		
3+ chronic diseases *v.* less (0–2)					*p* ⩽ 0.001	*p* ⩽ 0.001
Proportion 3+ [*n* (%)]	69 (19)	57 (29)	40 (39)^a^	35 (43)^a^		
Odds Ratio*	1	2.88	4.11	4.53		
(95% CI)		(1.27–6.48)	(1.71–9.85)	(1.82–11.30)		
Quality of life						
Fair/poor (4+) *v.* good or better (3−)					*p* ⩽ 0.005	*p* ⩽ 0.005
Proportion fair/poor [*n* (%)]	33 (9)	26 (13)	14 (14)	20 (24)^a^		
Odds Ratio*	1	1.62	1.60	3.46		
(95% CI)		(0.92–1.62)	(0.80–3.22)	(1.79–6.70)		

*Odds ratios are presented from the adjusted model.

a Significantly different from the group without OSA in unadjusted model.

**Fig. 3.**
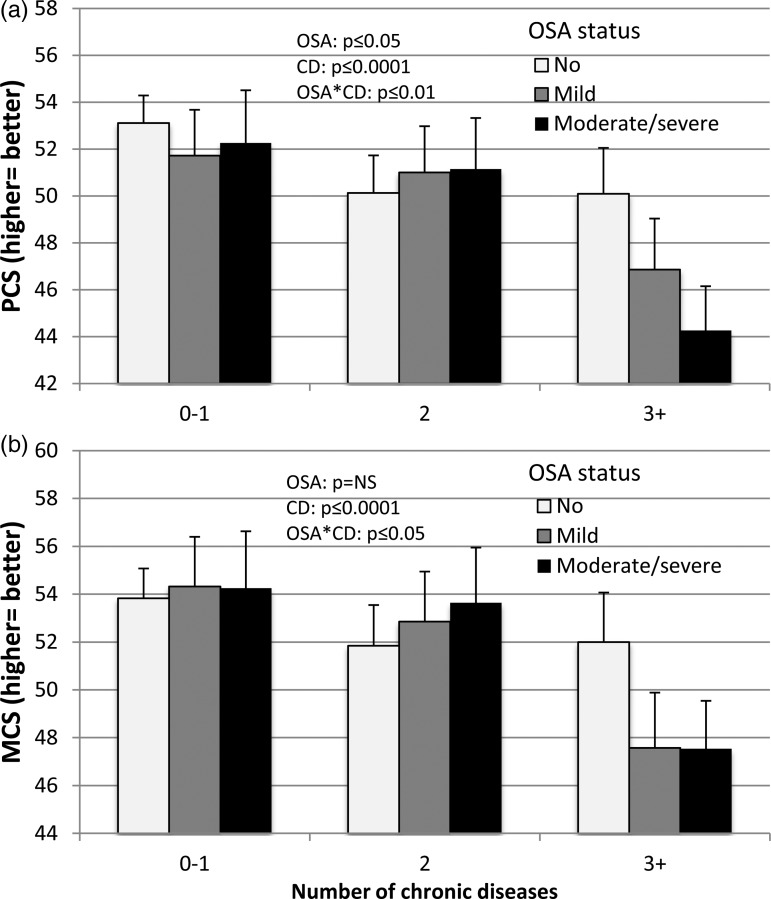
Interaction between obstructive sleep apnea and chronic disease count on physical (panel *a*) and mental (panel *b*) components summary of the SF36 quality of life questionnaire in the 743 men. CD, chronic disease; OSA, obstructive sleep apnea; PCS, physical component summary; MCS, mental component summary.

The interaction between multimorbidity and OSA severity status was significant for both the PCS and the MCS QoL measures (Fig. 3). For those with two or less CDs, there were no significant differences between OSA groups in both PCS and MCS mean values. However, in those with three or more CDs, PCS significantly decreased with OSA severity from 0.38 SD for mild to 0.68 SD for moderate/severe groups (*p* ⩽ 0.05 for moderate/severe *v.* mild). In contrast, there was a significant 0.50 SD decrease in MCS score in those with OSA, independently of the severity, than in those without. In the MCS model, while the interaction between OSA and multimorbidity was significant (*p* ⩽ 0.05), the effect of OSA status alone was not (p = ns) in opposition to the effect of multimorbidity (*p* ⩽ 0.001). A similar relationship as for MCS was found in those with three CDs for general health status (SF1).

## Discussion

The present study contributes to the understanding of the relationship between multimorbidity and OSA severity in previously undiagnosed men. More precisely, this study reports that in men over 40 years old without a prior OSA diagnosis, (1) multimorbidity is associated with OSA; (2) the association between multimorbidity and OSA severity is more important in those with more than three CDs; and (3) a detrimental interaction between multimorbidity and OSA on QoL was found and was associated with OSA severity in PCS.

A previous study investigated the relationship between OSA and multimorbidity, as measured by CD count and the disease burden multimorbidity assessment (DBMA), a composite index taking into account the self-reported presence and burden of different CDs in patients who had undergone PSG following referral by a physician [21]. Supportive of the results of the present study, *Robichaud-Halle et al.* reported an association between multimorbidity (DBMA) and severe OSA only [21]. The mean CD count was not significantly different between OSA severity status, which is in contrast to our findings of a progressively significant increase in the number of CDs through OSA severity status. A major difference and a strength of the present study is that it explored previously undiagnosed OSA cases, which represent the majority of those with OSA [37]. While the referral/diagnostic bias (OSA suspicion *v.* undiagnosed) could explain discrepancies, other differences such as the number of CDs and AHI cut-off should be acknowledged. Also, the AHI cut-off used to classify severity status in the Robichaud-Halle study was based on the 3% desaturation criteria to define hyponeas with OSA defined by an AHI ⩾5 while the present study used 3% and AHI ⩾10. Notwithstanding, they are roughly equivalent according to the Rheuland study [26] which was adopted by the AASM and has been shown to be associated with worst cardiovascular outcomes [26, 38, 39].

Another important finding of the present study is that while multimorbidity is associated with undiagnosed OSA, those with exactly two CDs are not at an increased risk of OSA. The proportion of those with three or more CDs increased with positive OSA status but also with severity status making the use of three CDs a more interesting cut-off than the two CDs generally used in multimorbidity to investigate OSA status but also severity as previously suggested [14].

The present results support the detrimental impact of multimorbidity [40] and OSA [41] on QoL. The potential relationship between OSA and multimorbidity on QoL was previously suggested but not assessed [21]. To the best of our knowledge, this study is the first to report a detrimental significant interaction effect between multimorbidity and OSA on QoL as measured by the SF-36 summary component scores. The impact of OSA was limited in those with two or less CDs although it became very important in those with more than three CDs. In this group, the presence of even mild OSA had an important detrimental impact on both the MCS and PCS scores. Interestingly, for PCS, the detrimental impact of OSA increased with its severity but this was not the case for MCS. In those with more than three CDs, the presence of undiagnosed OSA, even with mild severity, has an important impact on their QoL. Therefore OSA may be particularly important to identify in this population as, in clinical trials, continuous positive airway pressure (CPAP) therapy had a beneficial impact on PCS and MCS in most studies [42–44] but not all [45].

In a recent meta-analysis, an important bias noticed in the majority of analysed studies on OSA is that cohorts are mostly composed of patients tested on clinical suspicion of OSA and that information on undiagnosed cases are lacking [37]. The present study directly addresses this knowledge gap by investigating this population. Potential bias from those not participating in the study is possible but most likely accounted for by the relatively high proportion of eligible participants taking part in the PSG testing (59%). This is particularly important when considering that OSA is a condition with high undiagnosed rates and its timely treatment, could improve the management of other CDs [46] and improve health and QoL outcomes [38, 39, 47].

Strengths of the study include information on OSA severity (AHI), rather than OSA status and our large population (*n* = 743). This study has some limitations. While data have been adjusted for many risk factors, some potential residual confounders could still not be taken into account. The number of CDs investigated in the present study (9) is relatively small when compared with some other multimorbidity studies [14] and future studies should include a higher number of CDs. In addition, not including men with incomplete details on their CDs could have introduced bias. However, the diagnosis of most of the CDs used in the study is based on biomedical measurement or validated questionnaires which are more precise than most other data sources since it permits accounting for undiagnosed conditions which have been previously shown to be high for some CDs [48].

In summary, to the best of our knowledge, this study is the first to report an association between multimorbidity and OSA in a population of middle-aged to elderly men with undiagnosed OSA. This study also demonstrated that undiagnosed OSA has a major detrimental impact on the QoL of people with multimorbidity. Our data suggest that primary care physicians who treat men with multiple, chronic conditions should investigate the presence of OSA given its high rate of under diagnosis in the community and additional synergistic QoL burden.

## Contributors

AWT, GAW, SA and RJA designed the study and SAM coordinated the study. GR took overall lead in the statistical analysis of the data. All authors contributed to the interpretation of the data, writing of the report and approved the final manuscript.
